# Genealogical structure of the Colombian Romosinuano Creole cattle

**DOI:** 10.1007/s11250-023-03694-1

**Published:** 2023-08-17

**Authors:** Jhon Jacobo Cañas–Álvarez, Gustavo Alfonso Ossa-Saraz, Jorge Luis Garcés-Blanquiceth, William Orlando Burgos–Paz

**Affiliations:** 1 Corporación Colombiana de Investigación Agropecuaria-Agrosavia, Centro de Investigación Motilonia, Km 5 Vía Becerril, Cesar, Agustín Codazzi, Colombia; 2 Corporación Colombiana de Investigación Agropecuaria-Agrosavia, Centro de Investigación Turipaná, Km 13 Vía Montería-Cereté, Córdoba, Colombia; 3 Corporación Colombiana de Investigación Agropecuaria-Agrosavia, Centro de Investigación Tibaitatá, Km 14 Vía Mosquera-Bogotá, Cundinamarca, Colombia

**Keywords:** Effective population size, Genealogy, Genetic diversity, Inbreeding

## Abstract

The Romosinuano cattle breed is one of the most important Creole genetic resources in Colombia, and interesting traits like adaptation or reproductive efficiency have promoted its use in different countries in America. To consolidate the genealogical historical records, the review of very first yield records in this population was used to reconstruct the genealogy of the breed since the first animals incorporated to the in vivo germplasm bank and estimate different demographic parameters. The complete genealogy comprises 17,136 animals with 5.8 years of generation interval for two pathways. The estimated average inbreeding for the population and inbred animals was 2.53% and 6.32% respectively, with a progressive increase of inbred animals across the generations. Almost 48% of the total animals presented some level of consanguinity. Effective population size (Ne) based on the inbreeding rate estimated by regression in all generations was 120 animals whereas Ne estimated by equivalent generations was 69 animals. Effective number of founders (Fe), effective number of ancestors (Fa), and ancestors explaining 50% of variability were 75, 48, and 22, respectively. The relation between Fa/Fe of 64% indicates a genetic bottleneck effect in the population studied.

**Supplementary Information:**

The online version contains supplementary material available at 10.1007/s11250-023-03694-1.

## Introduction

The origin of the Romosinuano cattle (ROM) is traced to the very first cattle introduced to America, with a close relationship with Iberian cattle (Ginja et al. 2019). This hornless breed (“romo” is the term used for this phenotype in Colombia) has been traditionally maintained in the north coast of Colombia, particularly in the “Sinú Valley,” from where it adopted its name. Nowadays, ROM is the most important Creole beef cattle in Colombia due to adaptation to the tropical environment and the meat quality production (Flórez et al. 2014).

The very first government effort to promote the ROM conservation was the establishment of the breed germplasm bank in 1936, with 258 females (42.6% individuals belong to “El Torno” herd) and 12 males from different breeders (Ossa et al. 2013). Once the herd was created, a productive and genealogical data recording program was implemented. By 1963, the entire herd was transferred to the Turipaná Research Centre where it is maintained until today.

Since 1994, a permanent monitoring of diversity was implemented using the circular mating system (Martínez et al. 2008). Recently, some individuals from the germplasm bank have been selected to establish a genetic improvement program where growth and reproductive traits have been used as selection criteria (Ossa et al. 2008). Genealogical and productive information collected from 1980 has supported the genetic diversity management as well as the genetic evaluations, that alongside performance testing of young bulls has increased the inventory of this breed in Colombia.

In 2010, historical herd records were recovered from the AGROSAVIA files (Ossa et al. 2013) containing the very first herd records of the population. This data allowed to connect the pedigree until its conformation in 1936. However, issues like different animal identification patterns or discrepancies between dates limited the use of this data until today.

Because of the relevance of the pedigree data for support either genetic information of the herd or developing new strategies for animal selection, the objective of this work was to validate the individual relationships and reconstruct the pedigree data in order to revisit the genetic diversity and demography status of Colombian Romosinuano Creole cattle breed.

## Materials and methods

### Genealogical data preparation

The ROM population is maintained in the Turipaná Research Centre of the Corporación Colombiana de Investigación Agropecuaria, AGROSAVIA (www.agrosavia.co), located at the Sinú Valley in Cereté, Córdoba (8°50′15.2″ North and 75°47′33.9″ West). The center is around 14 m above sea level with average annual temperature of 28 °C, relative humidity ranged from 79 to 87%, and precipitation of 1200 mm.

Pedigree and productive performance were continuously updated since 1980, but previous pedigree records were only available in herd recording cards. Therefore, the first step was to include genealogical, growth, and reproductive data from cards into the breed database. Subsequently, a random sample of 10% of cards were reviewed manually to check typing errors or miscoding data.

Furthermore, pedigree records review was performed with Python and R (R Core Team, 2020) scripts that validated parentage relationships, bisexualities, and other mistakes in the pedigree consolidation. Main routine of data validation included (1) coherence between individual date of birth and weights at different ages; (2) validation age of dam at individual birth date, considering at least 22 months dam age at first parity; and (3) validation dam parity interval ranged from 380 to 440 days. Because some bulls were sire at most 14 years, special attention was required to validate heifer incorporation in the pedigree. Finally, historical data validation allowed to obtain 17,136 pedigree records between 1936 and 2019.

### Pedigree analyses

Once pedigree relationships, birth dates, and concordance between age of dams and parities were validated, numerous demography, structure, and genetic status parameters based on pedigree information were estimated:

#### Breed censuses

Correspond to the number of sires and dams with offspring in a given year. The parents of animals born in a given year are entered into the equation used to calculate the effective population size (${N}_{e}$) for each reporting year according to Falconer and Macky (1996):

$$Ne=\frac{4{N}_{m}{N}_{f}}{{N}_{m}+{N}_{f}}$$

where ${N}_{m}$ and ${N}_{f}$ are the number of male and female parents, respectively. The above formula refers to the number of breeding males and females in a population with discrete generations.

#### Generation interval

Defined as the average age of parents at the birth of their progeny kept for reproduction (James 1977), and the average age of parents at the birth of their offspring (used for reproduction or not). Both parameters were computed for the 4 pathways (father-son, father-daughter, mother-son, and mother-daughter).

#### Pedigree completeness level

Estimated as the average of MacCluer’s index (MacCluer et al. 1983) considering 1 to 6 generations deep. Additionally, the mean of maximum generations, complete generations, and the equivalent generations was estimated. The first is the number of generations separating the individual from its furthest ancestor. The second is defined as those separating the offspring of the furthest generation where the 2^ g^ ancestors of the individual are known. Ancestors with unknown parent were considered founders (generation 0). The equivalent generations were computed as the sum over all known ancestors of the terms computed as the sum of $\left(1/2\right)n$ where $n$ is the number of generations separating the individual to each known ancestor (Boichard et al. 1997).

#### Inbreeding coefficient and co-ancestry

The inbreeding coefficient (*F*) is the probability of having two genes identical by descent (Wright 1922). Moreover, the coefficient of inbreeding (*F*) of an individual is equal to the coefficient of co-ancestry (*f*) between its parents. Thus, the co-ancestry is defined as the probability that a progeny of 2 parents carries 2 alleles identical by descent. Under random mating, the rate of inbreeding (Δ*F*) equals the rate of co-ancestry (Δ*f*).

#### Effective population size (${N}_{e}$)

Was calculated using ${N}_{e}=1/\left(2\Delta F\right)$ whereas the rate of inbreeding per generation $\left(\Delta F\right)$ was calculated using $\Delta F=\left({F}_{n}-{F}_{n-1}\right)/\left(1-{F}_{n-1}\right)$, where ${F}_{n}$ and ${F}_{n-1}$ were the average inbreeding of offspring and their parents respectively (Falconer and Macky 1996). An alternative way to estimate ${N}_{e}$ is by computing the increase of inbreeding for each individual $\left(\Delta {F}_{i}\right)$ that is equal to $1-{\left(1-{F}_{i}\right)}^{1/t}$, in which ${F}_{i}$ is the inbreeding coefficient (González-Recio et al. 2007) and *t* can be, for each individual, the equivalent generations, the maximum number of generations known, or the complete generations (Gutiérrez et al. 2008). By averaging these $\Delta {F}_{i}$, an estimate of $\Delta F$, $\acute{\Delta F}$ in the reference population can be obtained, and then a realized ${N}_{e}$ is computed as $\acute{{N}_{e}}=1/2\acute{\Delta F}$ (Cervantes et al. 2008).

#### Effective number of founders (${f}_{e}$)

Was obtained as the number of equally contributing founders that would be expected to produce the same genetic diversity as in the population under study (Lacy 1989). It was calculated as ${f}_{e}=1/{\sum }_{k=1}^{f}{q}_{k}^{2}$, where ${q}_{k}$ is the probability of gene origin of the k ancestor, that is, the proportional contribution of founder k. When founders contribute unequally, the effective number of founders is smaller than is the actual number.

#### Effective number of ancestors

Unlike the effective number of founders, this parameter considers possible bottlenecks in the population, which is a major cause of gene loss in cattle populations. It is the minimum number of ancestors (founders or not) necessary to explain the complete genetic diversity of the population under study (Boichard et al. 1997) and is computed as ${f}_{a}=1/{\sum }_{j=1}^{a}{q}_{j}^{2}$. In this case, ${q}_{j}$ is the marginal contribution of the *j* ancestor, that is, the genetic contribution made by an ancestor that is not explained by other ancestors chosen previously.

The estimation of demographic parameters was performed with POPREP (Groeneveld et al. 2009) and ENDOG (Gutiérrez and Goyache 2005) software. POPREP was used to compute the census trend, family sizes, inbreeding, co-ancestry, and ${N}_{e}$ calculated from the last 2 parameters, whereas ENDOG was used to calculate the average pedigree completeness, generation interval, maximum, complete and equivalent generation, individual increase of inbreeding, and the corresponding realized ${N}_{e}$ and probabilities of gene origin: effective number of founders and effective number of ancestors.

## Results and discussion

### Pedigree information and relatedness

**Fig. 1:**
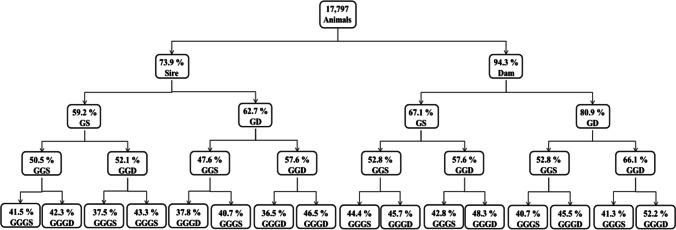
Pedigree completeness of the Romosinuano population

Until 2020, pedigree information of ROM cattle included 5667 individual records since 1980. This data has been used for estimation of genetic parameters of different productive traits (Ossa et al. 2008; Vergara et al. 2016). When historical data was validated and organized, the pedigree increased to 17,797 individuals. Out of this, 16,782 (93.4%) individuals have dam record whereas the 73.9% have the sire record (Fig. 1). Until fourth generation, the pedigree completeness was 42.43%, mainly for the maternal lineage.

Due the herd management and ROM behavior, identification of dams became easier than sires. Additionally, the exceptional libido of the Creole sires (Chenoweth et al. 1996; Armstrong et al. 2022) might result in either non-correct pedigree assignations or lack of information. The average maximum number of generations was 7.9, and the equivalent complete generations were 3.91, similar to other Creole cattle breed in Colombia (Martínez et al. 2008) like Costeño con Cuernos (3.70) and Sanmartinero (3.80). The average complete generations in ROM were 1.73, similar to those observed in Spanish autochthonous breeds (Cañas-Álvarez et al. 2014).

**Fig. 2:**
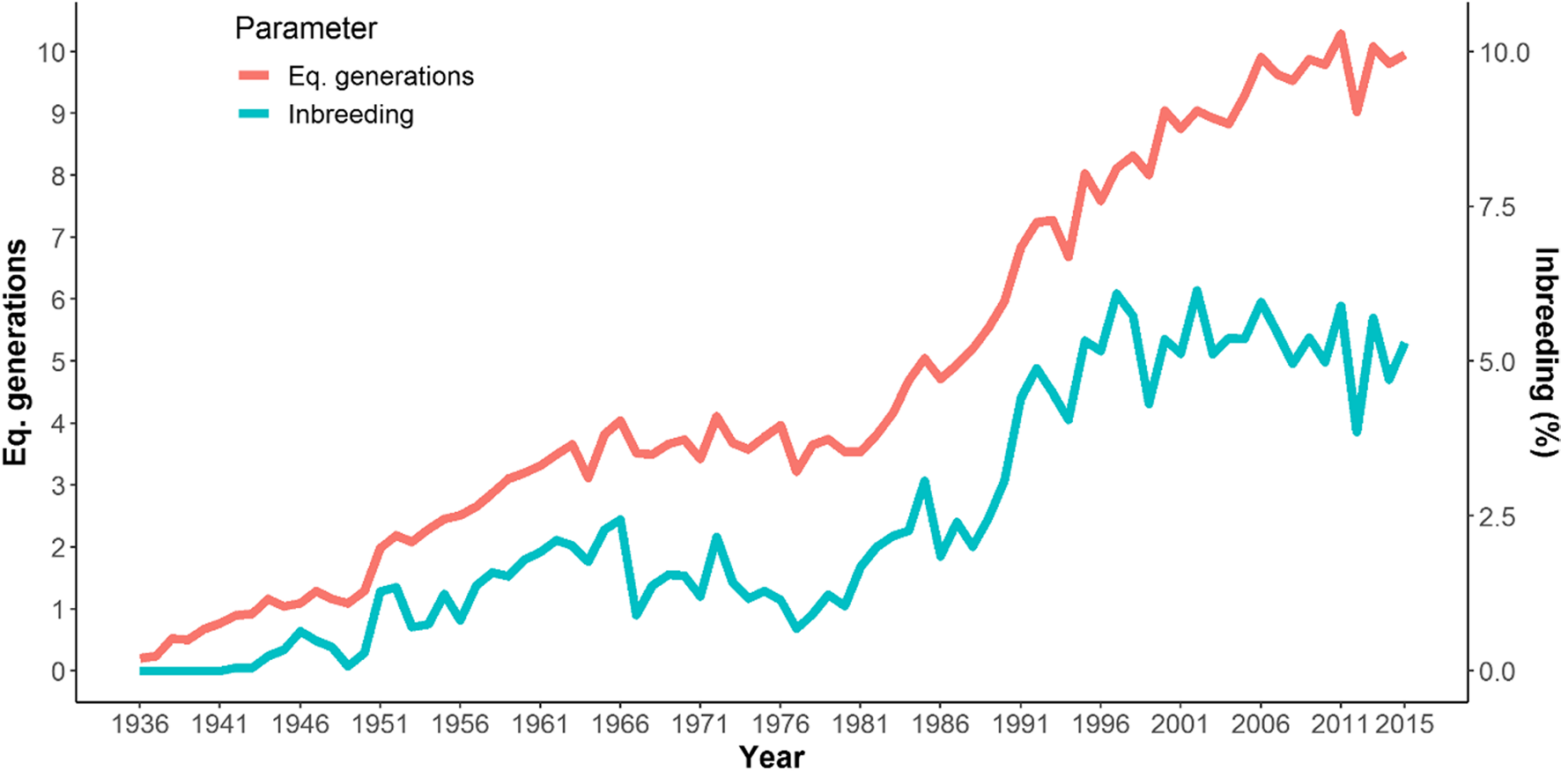
Evolution of equivalent generations and mean inbreeding coefficients across years

Figure 2 shows the evolution of the equivalent generations from 1936 to 2018. Although the average of equivalent complete generations was 3.91, this parameter steadily increased after 1980, reaching 10 generations by 2015. This could be attributed to the pedigree data control for genetic evaluation. Despite this trend, it is important to interpret the equivalent complete generations with caution, particularly when considering the newly detected relationships. For instance, some values for equivalent complete generations (9.3) and fourth generation known ancestors (90%) observed in other cattle breeds are desirable (Gutiérrez et al. 2003; Bouquet et al. 2011), but in a small population, a relation of this parameter with inbreeding coefficient must be considered (De La Rosa et al. 2016).

When the number of equivalent generations was related with the average value of inbreeding, inclusion of historical data resulted in a slight increase of inbreeding in ROM regarding to previous estimations (Martínez et al. 2008). The increase of pedigree revealed the intensive use of particular bulls, even for about 14 years between end of the 50’s to early 70’s decades. As expected, the small size of herd caused a sustained increase of consanguinity since 1936, but in the 90’s decade, the inbreeding reached a plateau and the last 6 years average inbreeding was around 5% (Fig. 2). The circular mating (Nomura and Yonezawa 1996) and low inbreeding individuals mating in ROM population have made an important contribution to maintaining this parameter as low as possible.

### Romosinuano population size

**Fig. 3:**
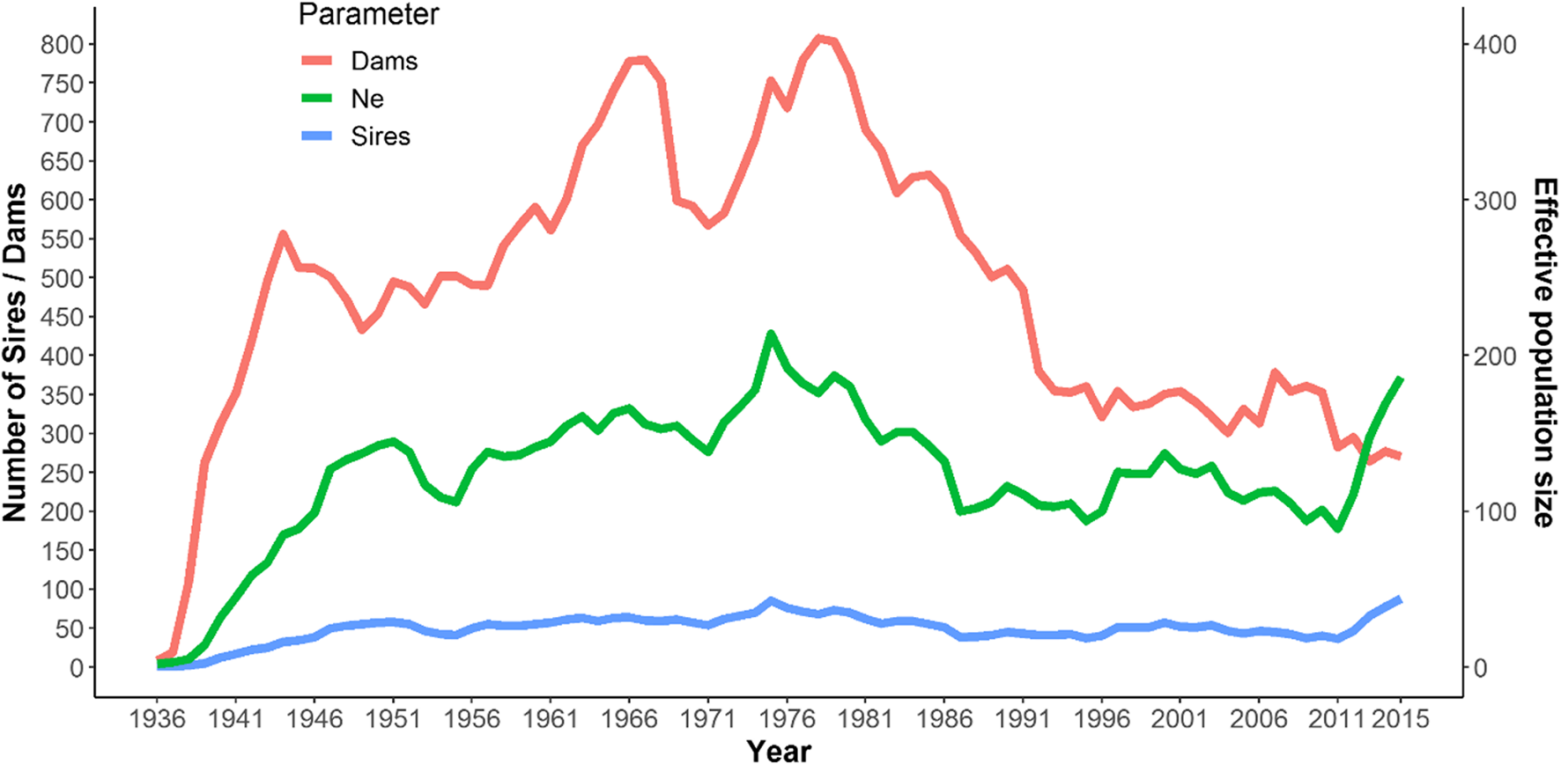
Evolution of number of dams, sires, and effective population size across years

Figure 3 illustrates the number of breeding males and females in the ROM population with discrete generations. A generation of animals corresponding those animals born in the time span of one generation interval (GI) window with ends in the year reported in the figure and their of each GI window were employed to compute the effective population size (Ne) per year.

During the initial attempt to establish a ROM herd, individuals were collected from few herds, potentially leading to the inclusion of closely related or inbred individuals among the founders (Ossa et al. 2013). Furthermore, population increase was a priority during the first years of the herd (Fig. 1). The applied breeding strategies resulted in different constrains over the years like the use of bulls for over 14 years with a large number of progeny (Supplementary file, Table S1) with the subsequent inbreeding increase, or the use of ROM dams for crossbreed evaluation with Zebu cattle (Hernandez 1976; Mueller-Haye and Gelman 1981).

The number of dams per sire has decreased across the years, where at 1938 it was 54 whereas in 2015 it was only 3.1 dams/sire. Between 1980 and 1990, a remarkable decrease in the number of dams was observed due to difficult environmental conditions (forage availability reduction and drought) alongside economic limitations that increased the environmental impact.

Since 2005, well-established strategies including seminal conservation in the germplasm bank, performance test, and planning mating recommendations for ROM breeders promoted the use of the breed.

Currently, Estimated Breeding Values (EBVs) from genetic evaluation and inbreeding coefficients are employed to select individuals for mating, considering higher EBVs for growth traits, lower EBVs for calving interval, and inbreeding values below 0.04 (Vergara et al. 2016).

For the maintenance and improvement of conserved populations, Food and Agriculture Organization FAO (1998) recommends having a minimum Ne of 50 animals, which was higher in ROM except for the first 4 years (1936–1940). The effective population size in the last 5 years increased mediated by a higher number of sires in the herd.

### Generation interval

The average GI for each of the four pathways is summarized in Table 1. The interval by maternal pathway was superior in almost 1.5 years with respect to the paternal pathway, because the dams remain in the herd longer than males and the later are quickly replaced.

**Table 1:** Generation interval estimates for the four pathways and average age in years of sires and dams

	***N***	Value	Standard error
Interval father-son	324	4.76	0.10
Interval father-daughter	2431	5.01	0.04
Interval mother-son	356	6.28	0.15
Interval mother-daughter	2803	6.36	0.05
Total interval	5914	5.71	0.03
Age father-son	5527	5.14	0.03
Age father-daughter	5616	5.20	0.03
Age mother-son	7224	6.37	0.04
Age mother-daughter	7270	6.34	0.04
Total age	25637	5.84	0.02

The observed GI was lower than reported in foreign breeds like Charolais, Limousin, Hereford, Angus, Shorthorn, and Simmental (Herron and Pattie 1977; Mc Parland et al. 2007), and even lower compared to Spanish autochthonous breeds Avileña-Negra Iberian, Bruna dels Pirineus, Morucha, Pirenaica, Retinta, and Rubia Gallega (Cañas-Álvarez et al. 2014). In comparison with other Colombian Creole breeds, the GI was similar to Costeño con Cuernos (5.4 years) and intermediate to Blanco Orejinegro (4.7 years) and Sanmartinero (6.8 years) breeds (Martínez et al. 2008).

The average age of parents at the offspring birth was similar to GI showing that most of the animals (specially females) were used for reproduction. Due the low census of ROM, selection pressure has been limited to males while almost all females are retained for calving. Family size estimates of breeding animals per generation are presented in Table S2 (Supplementary file). The progeny per sire in all the offspring born in the population varied from 9.9 to 78.9 in generations 12 and 2, respectively, which is again related to continued use of same bulls for many years in the first generations. Similar trend was observed in dams.

### Inbreeding and effective population size

The essential consequence of two individuals having a common ancestor is that both have one of the genes present in the ancestor. This means that inbreed individuals carry two genes in a locus that are replicas of a single gene in a previous generation (Falconer and Macky 1996). Table 2 shows the number of records, the highest consanguinity, the average consanguinity and its deviation, the number of consanguineous animals with their percentage, and the average of the consanguinity of said animals and their deviation from the individuals belonging to each evaluated generation.

**Table 2:** Average inbreeding coefficients of all animals and inbred animals born in each generation

Generation	Period	All animals			Inbred animals	
		Number	Maximum $F$	Average $F$ (SD)	Number (%)	Average $F$ (SD)
1	1938–1943	208	0.1250	0.0002 (0.0026)	1 (0.48)	0.1250 (0.00)
2	1944–1949	254	0.3125	0.0036 (0.0233)	6 (2.42)	0.1459 (0.0564)
3	1950–1955	246	0.2500	0.0094 (0.0272)	37 (15.07)	0.0648 (0.0417)
4	1956–1961	299	0.2578	0.0151 (0.0337)	102 (34.19)	0.0510 (0.0473)
5	1962–1967	304	0.2617	0.0192 (0.0339)	177 (58.31)	0.0331 (0.0392)
6	1968–1973	249	0.2813	0.0154 (0.0314)	125 (50.17)	0.0304 (0.0387)
7	1974–1979	280	0.2790	0.0107 (0.0247)	116 (41.27)	0.0263 (0.0336)
8	1980–1985	244	0.3225	0.0204 (0.0413)	107 (43.84)	0.0449 (0.0524)
9	1986–1991	174	0.3092	0.0270 (0.0368)	99 (57.16)	0.0457 (0.0389)
10	1992–1997	149	0.2738	0.0500 (0.0427)	118 (79.60)	0.0631 (0.0385)
11	1998–2003	136	0.2833	0.0530 (0.0346)	111 (81.74)	0.0655 (0.0257)
12	2004–2009	147	0.1382	0.0542 (0.0279)	127 (86.38)	0.0629 (0.0187)
13	2010–2015	107	0.3014	0.0507 (0.0331)	86 (80.03)	0.0635 (0.0235)
Average		215	0.3225	0.0253 (0.0302)	93 (48.51)	0.0632 (0.0379)

Noteworthy, a progressive increase of the percentage of inbred animals to the total of animals was observed, reaching 80% (out 86 of 107 individuals) at generation 13 (Table 2). In the last generation, the average inbreeding estimated for inbred animals was *F* = 0.063, higher to the inbreeding observed for the entire population (*F* = 0.050). The genealogical information recovered is important for a better comprehension of individual relationships, particularly for the conservation program of the breed

The average inbreeding coefficient for all the animals increased over the years with values between 0.02% in the first generation and 5.42% in the 13th generation (Table 2 and Fig. 1). The maximum consanguinity in the population ranged between 12.5 and 32.25% (in the first and eighth generation, respectively) and it reflects mating of half-sibling parents or full siblings in the second generation (Supplementary file, Table S3). The inbreeding coefficient is probably the most important parameter in the analysis of the genetic structure, since it measures the probability of homozygosis in a genealogy and it is precisely homozygosity that causes inbreeding depression (Cervantes et al. 2008) and the additive genetic variance reduction within populations (Kristensen and Sorensen 2005).

The Ne based on the increase in inbreeding varied from 21 to 1000 for the tenth and first generation, respectively, while the Ne based on co-ancestry was higher, with a range of 23 to 1587 for the same generations (Table S4). The estimates of Ne in Supplementary file, Table S4 are much lower than those presented in Fig. 3, and deviates from an idealized population where sires and dams can mate at random. In some cases, the increase in inbreeding and co-ancestry was negative; for this reason, the effective size could not be calculated.

When the information of the pedigree is non-existent or incomplete, the Ne can be overestimated, particularly if the calculation is considered an extended period (De Rochambeau et al. 2000; Cassell et al. 2003). The result obtained for Ne of 169 and increase of consanguinity of 0.003 according to the maximum number of generations is an indicator of how the estimation of this type of factors can be strongly influenced by the depth of the pedigree (Table 3). Considering the equivalent number of generations, we can have a value adjusted for the effective population size, which is 69 animals for the ROM population. This value was found among the majority of Spanish breeds (Cañas-Álvarez et al. 2014).

**Table 3:** Average of inbreeding and effective population sizes (${N}_{e}$) computed and its increase ($\Delta F$) in the animals

	$\Delta F$	${N}_{e}$
By maximum generations	0.0030	169
By complete generations	0.0146	34
By equivalent generations	0.0073	69

Finally, the probability of the gene origin in ROM population analysis showed that the effective number of founders (fe), the effective number of ancestors (fa), and the number of ancestors that explain 50% of the genes were 75, 48, and 22 respectively. The three parameters in ROM were higher than the average of four Creole Colombian breeds (74.6, 41, and 16, respectively) reported by Martínez et al. (2008). The recent increase of amount of ROM genealogy information (reference population 13,129 individuals) as well the pedigree depth contributed in these differences. The increase in the parameters is desirable considering that the limited size of the populations and the several efforts to maintain the variability.

For instance, the genealogical information showed that ROM population fluctuated in size and breeding scheme since 1936 (Table S2-S4, supplementary file) and the relation of ancestors and founders (fa / fe = 0.64) revealed that the minimum number of ancestors required for the maintenance of the genetic variability was low. Some sires and dams were kept in the herd for more than 10 years, reducing the population diversity and generating bottlenecks. The fa/fe proportion closer to 1 indicates no genetic bottleneck in the population, but if proportion decreases the impact is greater (Boichard et al. 1997).

For Creole cattle populations like Lageana from Brazil (Pezzini et al. 2018), Limonero from Venezuela (Villasmil-Ontiveros et al. 2008), or Blanco Orejinegro from Colombia (Ocampo et al. 2020), the reported number of ancestors that explain 50% of genetic diversity was 10, 18, and 14 respectively. Here, we traced around 80 years of pedigree history and the number of ancestors that explain 50% of genetic diversity in ROM can be considered low.

## Conclusions

The increase in genealogical information within the Romosinuano population has proven valuable in estimating inbreeding coefficients, effective population size, and pedigree completeness. These parameters have provided insights into the Romosinuano demographic evolution, revealing the impact of bottlenecks and the intensive use of bulls on the effective population size. The inclusion of deep pedigree relationships showed a slightly inbreeding levels increase. To mitigate potential effects of inbreeding on productivity traits, it is essential to monitor mating based on new co-ancestry estimations. Furthermore, the new pedigree should be evaluated with phenotypic traits to recalculate the genetic parameters associated with these traits and the relation of inbreeding and performance.

### Supplementary Information

Below is the link to the electronic supplementary material.

Supplementary file1 (DOCX 18 KB)

## Data Availability

The datasets analyzed during the current study are available from the corresponding author upon reasonable request.
