# Supplementary material for: Genealogical structure of the Colombian Romosinuano Creole cattle
Source: Trop Anim Health Prod. 2023 Aug 17;55(5):292. doi: 10.1007/s11250-023-03694-1 (PMC10435628; doi:10.1007/s11250-023-03694-1)
Supplement: Supplementary file 1 — Supplementary file1 (DOCX 18 KB) [file 11250_2023_3694_MOESM1_ESM.docx]

**Supplementary material**

**Genealogical structure of the Colombian Romosinuano creole cattle**

Jhon Jacobo Cañas–Álvarez^1^, Gustavo Alfonso Ossa-Saraz^2^, Jorge Garcés-Blanquiceth^2^, William Burgos–Paz^3*^

^1^ Corporación Colombiana de Investigación Agropecuaria- Agrosavia, Centro de Investigación Motilonia, Km 5 vía Becerril, Agustín Codazzi, Cesar, Colombia.

^2^ Corporación Colombiana de Investigación Agropecuaria-Agrosavia, Centro de Investigación Turipaná, Km 13 vía Monteria-Cereté, Córdoba, Colombia.

^3^ Corporación Colombiana de Investigación Agropecuaria-Agrosavia, Centro de Investigación Tibaitatá, Km 14 vía Mosquera-Bogotá, Cundinamarca, Colombia.

* Corresponding author: [wburgos@agrosavia.co](mailto:wburgos@agrosavia.co)

**Table S1.** Sires with the most Progeny and the most Selected Progeny in the ROM breed

| **ID-numbers of 20 Sires with the most Progeny** | **Number of Progeny per Sire** |  | **ID-numbers of 20 Sires with the most Selected Progeny** | **Number of Selected Progeny per Sire** |
| --- | --- | --- | --- | --- |
| RT.908 | 266 |  | RT.908 | 66 |
| RT.1003 | 174 |  | R88071 | 44 |
| RT.1999 | 148 |  | RT.1003 | 43 |
| R62009 | 132 |  | R68037 | 40 |
| R68037 | 130 |  | R62009 | 32 |
| RT.1645 | 112 |  | RT.1645 | 31 |
| RT.564 | 106 |  | R87035 | 30 |
| R63185 | 102 |  | R81009 | 29 |
| RT.719 | 102 |  | RT.15 | 29 |
| RT.1803 | 98 |  | R74197 | 28 |
| RT.1559 | 96 |  | RT.1999 | 28 |
| R72111 | 95 |  | R75415 | 27 |
| R80031 | 94 |  | R80031 | 27 |
| RT.570 | 94 |  | RT.570 | 26 |
| RT.2102 | 94 |  | R69279 | 26 |
| RT.2042 | 92 |  | RT.30 | 25 |
| RT.1830 | 90 |  | RT.1559 | 25 |
| R88071 | 90 |  | R69045 | 25 |
| RT.1006 | 90 |  | RT.564 | 24 |
| RT.575 | 89 |  | R63185 | 24 |

**Table S2**. Average family sizes of breeding animals born in each generation

| **Generation** | **Period** | **All offspring^1^** | |  | **Selected offspring^2^** | |
| --- | --- | --- | --- | --- | --- | --- |
|  |  | **Sires** | **Dams** |  | **Sires** | **Dams** |
| 1 | 1938-1943 | 19.8 | 6.9 |  | 7.6 | 2.1 |
| 2 | 1944-1949 | 78.5 | 6.1 |  | 26.5 | 2.1 |
| 3 | 1950-1955 | 44.7 | 5.6 |  | 10.7 | 1.9 |
| 4 | 1956-1961 | 41.8 | 3.9 |  | 8.6 | 1.6 |
| 5 | 1962-1967 | 37.5 | 4.5 |  | 10.0 | 1.8 |
| 6 | 1968-1973 | 44.5 | 3.9 |  | 14.5 | 1.8 |
| 7 | 1974-1979 | 29.1 | 3.5 |  | 9.7 | 1.6 |
| 8 | 1980-1985 | 28.4 | 3.2 |  | 10.8 | 1.8 |
| 9 | 1986-1991 | 25.9 | 4.3 |  | 10.5 | 2.3 |
| 10 | 1992-1997 | 17.9 | 4.3 |  | 6.1 | 1.8 |
| 11 | 1998-2003 | 22.1 | 4.4 |  | 6.0 | 1.8 |
| 12 | 2004-2009 | 9.9 | 3.2 |  | 4.5 | 1.2 |
|  | **Total average** | **28.0** | **4.4** |  | **9.2** | **1.8** |

^1^All offspring born in the population.

^2^Offspring that have at least a service record.

**Table S3.** Distribution of number of animals born by year and inbreeding levels

| **Generation** | **Period** | **Inbreeding levels** | | | | | | |
| --- | --- | --- | --- | --- | --- | --- | --- | --- |
|  |  | **0 %** | **0.1 – 5 %** | **6 – 10 %** | **11 – 15 %** | **16 – 20 %** | **21 – 25 %** | **≥ 26%** |
| 1 | 1938-1943 | 1293 | 0 | 0 | 2 | 0 | 0 | 0 |
| 2 | 1944-1949 | 1489 | 0 | 4 | 18 | 9 | 5 | 1 |
| 3 | 1950-1955 | 1251 | 106 | 79 | 32 | 0 | 5 | 0 |
| 4 | 1956-1961 | 1182 | 436 | 91 | 72 | 10 | 4 | 1 |
| 5 | 1962-1967 | 760 | 836 | 136 | 69 | 16 | 4 | 2 |
| 6 | 1968-1973 | 745 | 617 | 89 | 21 | 17 | 2 | 4 |
| 7 | 1974-1979 | 986 | 607 | 55 | 17 | 12 | 0 | 2 |
| 8 | 1980-1985 | 821 | 483 | 82 | 36 | 24 | 6 | 10 |
| 9 | 1986-1991 | 446 | 367 | 182 | 30 | 15 | 0 | 1 |
| 10 | 1992-1997 | 182 | 295 | 329 | 53 | 28 | 3 | 2 |
| 11 | 1998-2003 | 149 | 178 | 430 | 53 | 5 | 0 | 1 |
| 12 | 2004-2009 | 120 | 179 | 565 | 17 | 0 | 0 | 0 |
| 13 | 2010-2015 | 128 | 133 | 370 | 6 | 1 | 0 | 3 |

**Table S4.** Inbreeding ($\Delta F$), coancestry ($\Delta f$) rates and effective population size ($N_{e}$) by generation

| **Generation** | **Period** | $\Delta f$ | $N_{e}$ ***(***$\Delta f$**)** | $\Delta F$ | | $N_{e}$ **(**$\Delta F$**)** |
| --- | --- | --- | --- | --- | --- | --- |
| 1 | 1938-1943 | 0.00032 | 1587 | 0.00050 | | 1000 |
| 2 | 1944-1949 | 0.00061 | 820 | 0.00342 | | 146 |
| 3 | 1950-1955 | 0.00255 | 196 | 0.00580 | | 86 |
| 4 | 1956-1961 | 0.00860 | 58 | 0.00573 | | 87 |
| 5 | 1962-1967 | 0.00492 | 102 | 0.00420 | | 119 |
| 6 | 1968-1973 | -0.00109 | NE | -0.00388 | | NE |
| 7 | 1974-1979 | -0.00251 | NE | -0.00480 | | NE |
| 8 | 1980-1985 | -0.00155 | NE | 0.00980 | | 51 |
| 9 | 1986-1991 | 0.01026 | 49 | 0.00676 | | 74 |
| 10 | 1992-1997 | 0.02146 | 23 | 0.02361 | | 21 |
| 11 | 1998-2003 | 0.01399 | 36 | 0.00305 | | 164 |
| 12 | 2004-2009 | 0.00371 | 135 | 0.00123 | | 408 |
| 13 | 2010-2015 | -0.00073 | NE | -0.00367 | | NE |
|  | **Total** | **0.00489** | **102** | **0.00417** | **120** | |

NE = Not estimable.
